# Mental Health Liaison Program with Schools in Madrid

**DOI:** 10.1192/j.eurpsy.2025.125

**Published:** 2025-08-26

**Authors:** C. Arango

**Affiliations:** Hospital General Universitario Gregorio Marañon, UCM, CIBERSAM, Madrid, Spain

## Abstract

**Abstract:**

Mental health issues among children and adolescents have risen, particularly following the COVID-19 pandemic. Despite increased awareness, less than half receive necessary care, leading to long-term consequences. The World Health Organization advocates for integrated, preventive community interventions to address this gap. This paper presents the **Mental Health Clinical Liaison Programme for Schools** in the Community of Madrid, Spain, which provides school-based activities led by multidisciplinary mental health teams. The programme focuses on early detection, intervention, and prevention strategies for children and adolescents. We describe its implementation, review supporting evidence, provide preliminary data, and discuss its scope and challenges. Between 2023 and 2025, the programme has reached over 100 primary and secondary schools, identifying more than 1,700 cases, evaluating over 500 students, and referring 232 to specialized services. It has also supported interventions for more than 400 students already in mental healthcare and facilitated 45 reintegrations following psychiatric hospitalization. Additionally, anti-stigma workshops have engaged approximately 2,500 students. Ongoing research aims to assess the programme’s effectiveness and cost-effectiveness to ensure continuous improvement in mental health services for young people.

**Disclosure of Interest:**

None Declared

